# The Translatome Map: RNC-Seq vs. Ribo-Seq for Profiling of HBE, A549, and MCF-7 Cell Lines

**DOI:** 10.3390/ijms252010970

**Published:** 2024-10-12

**Authors:** Anna Kozlova, Elizaveta Sarygina, Ekaterina Ilgisonis, Svetlana Tarbeeva, Elena Ponomarenko

**Affiliations:** Institute of Biomedical Chemistry, 119121 Moscow, Russia; lapd@ibmc.msk.ru (A.K.);

**Keywords:** translatome, Ribo-seq, RNC-seq, proteome, sequencing, cell lines

## Abstract

Gene expression is a tightly regulated process that involves multiple layers of control, including transcriptional, post-transcriptional, and translational regulation. To gain a comprehensive understanding of gene expression dynamics and its functional implications, it is crucial to compare translatomic, transcriptomic, and proteomic data. The two most common analysis methods, Ribo-seq and RNC-Seq, were used to analyze the translatome of the same sample, whose datasets were downloaded from the TranslatomeDB database. The resulting translatome maps obtained for three cell lines (HBE, A549, and MCF-7) using these two methods were comparatively analyzed. The two methods of translatome analysis were shown to provide comparable results and can be used interchangeably. The obtained mRNA translation patterns were annotated in the transcriptome and proteome context for the same sample, which may become the basis for the reconstruction of the molecular mechanisms of pathological process development in the future.

## 1. Introduction

The integrative analysis of translatomic, transcriptomic, and proteomic data provides a powerful approach to unraveling the regulatory complexity of gene expression [1]. By comparing these datasets, researchers can validate transcriptional regulation networks, identify translational regulatory mechanisms, uncover functional insights, and improve gene annotation [2,3,4]. This multidimensional approach enhances our understanding of cellular processes, disease mechanisms, and the functional implications of gene expression at different levels [5,6].

Many studies have shown that there is no clear correlation between the transcriptome and the proteome [7,8]. The intermediate level of molecular organization between the transcriptome and the cell proteome is the translatome [9]—the complete set of mRNAs that are currently being translated into proteins in a cell or tissue at a given time. Not all mRNAs are actively being translated into proteins at any given time, and the composition of the translatome can vary depending on cellular conditions and the specific physiological state of the cell or tissue [10].

The first method used to study translation and the translatome is polysome profiling. The essence of this method is the separation of actively translated mRNAs bound by several ribosomes (polysomes), “free” RNA, small (40S) and large (60S) ribosomal subunits, and monosomes by centrifugation in a sucrose gradient (for the first mention of polysomal profiling and the recent protocol, see [11,12]) The distribution of specific mRNAs in gradient fractions can be monitored using classic RNA quantification methods (such as Northern blotting, RT-PCR, microarrays) or high-throughput sequencing [13]. Combining polysome profiling with RNA-seq is also referred to as the ribosome–nascent chain complex-bound mRNA sequencing or RNC-seq [14]. The RNC-seq method is time-consuming, and it does not allow for analyzing samples in parallel [12]. However, the analysis of RNC-seq data is quite similar to the analysis of RNA-seq data, since RNC-seq can obtain reads of different lengths depending on the sequencing platform, which increases the confidence in the detection of genes and splice forms.

Another approach of translatome studies is Ribo-seq or ribosome profiling. Ribo-seq is based on deep sequencing of short mRNA fragments (28–31 nt) physically enclosed by ribosomes [15]. Compared to RNC-seq in ribosomal profiling, translation is stopped by translation inhibitors, and thus, the current positions of ribosomes on mRNA are captured [16]. Recent Ribo-seq protocols can achieve single-nucleotide resolution with high reproducibility. Such resolution allows Ribo-seq to de novo identify mRNA open reading frame (ORF) regions and detect potential cryptic translation events [17]. However, the analysis of ribosomal profiling data is quite difficult because the RPFs obtained are quite short in length, which requires the optimization of bioinformatics data processing and filtering of false-positive gene detections.

In addition to Ribo-seq and RNC-seq, there are a number of other methods for studying insights into the translation process, for example, translation complex profiling sequencing (TCP-seq), which is commonly used for detecting translation initiation sites in ORFs [18], and translating ribosome affinity purification (TRAP), which is a tool for monitoring translation in specific cell types [19]. Methods based on immunoprecipitation, for example, CLIP-seq, which allows for revealing the profile of interactions of RNA with RNA-binding proteins, can also be effective in studying translation regulation [20].

Both the Ribo-seq and RNC-seq methods have a number of limitations associated with sample preparation and analysis of the obtained data. To carry out translatome profiling using the Ribo-seq or RNC-seq techniques, it is necessary to obtain a sufficient amount of biomaterial (at least 10^7^ cells), which can be quite difficult in the case of human native tissues. As mentioned above, both methods require proper bioinformatics approaches to process the raw data to avoid biases in the resulting translation profiles. Sequencing of short reads, which is used in Ribo-seq, is fraught with the production of multiple mapping reads, which reduces the accuracy of the quantitative and qualitative determination of the level of translation of specific genes.

It should be noted that the accurate identification of translated protein-coding genes in the translatome profile is important for performing meta-analyses using different omics levels, including the proteome. For this reason, it is very important to choose an appropriate translatome profiling method that provides the most complete coverage of the protein-coding genes of an organism, particularly in humans. Therefore, this work aims to compare the ability of two translatome profiling methods to characterize translation on an entire set of protein coding genes.

## 2. Results

### 2.1. Trends in the Application of Translatome Profiling Methods

**Figure 1:**
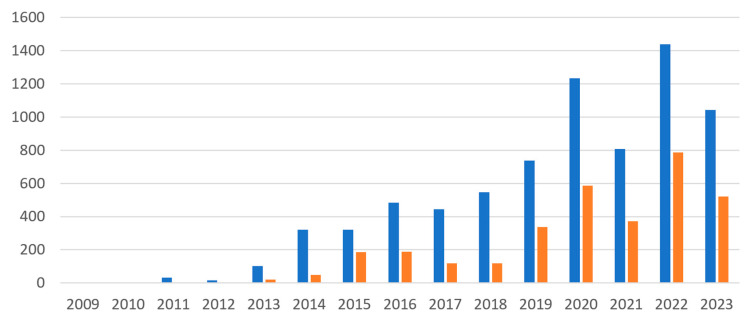
Accumulation of datasets in the GEO database on Ribo-seq. Blue bars represent the total number of datasets, and orange bars represent the number of human datasets.

In the GEO database, a query for ‘ribosome-profiling’ OR ‘riboseq’ OR ‘ribo-seq’ OR ‘ribosome profiling’ returned 7023 entries, of which 3097 were related to humans. This indicates the widespread use of translatome profiling methods in human studies. The first mention of these methods in the GEO database dates back to 2009 (Figure 1). Predictably, the number of non-human translatome datasets was greater than those for humans. The use of large-scale human samples for translatome analysis is often limited by ethical constraints, challenges in obtaining sufficient amounts of human tissue, difficulties in sample preparation, and higher associated costs. Also, while translatome profiling is gaining traction, particularly in oncology and personalised medicine, its adoption in human studies is still catching up with more traditional transcriptome approaches such as RNA-seq.

Table 1 contains the results of queries related to “Ribo-seq” and “RNC-seq” experiments conducted in various databases such as PubMed, PMC Full-Text, BioProject, GEO DataSets, and DB Translatome. From the results of the table, it can be concluded that the use of the RNC-seq method is much less frequent.

Analysis of the usage frequency of ribosomal (Ribo-seq) and polysomal (RNC-seq, Poly-seq) translatome analyses using a system for automatic processing of published data [21] revealed that Ribo-seq was used much more frequently. In the PubMed database, the query “Ribo-seq” returned 1454 publications (February 2024), with a decreasing trend in publications after 2021. The query “RNC-seq” or “Ribosome nascent-chain complex-bound mRNA sequencing” returned only 210 papers, i.e., ribosomal profiling is more popular than polysomal profiling.

According to TranslatomeDB [22], the number of datasets with the results of translatome analysis was 2456 for Ribo-seq and only 10 for RNC-seq (2017). By comparison, the number of datasets was as many as 4054 for Ribo-seq and 216 for RNC-seq in 2024.

### 2.2. Ribo-Seq and RNC-Seq Provide Comparable Gene Quantification

According to the data obtained, at least one transcript was detected by RNA-seq for each of 80% of human protein-coding genes (PCGs) at an RPKM (Reads Per Kilobase Million) cutoff of >0. This indicator was stable for the three analyzed cell lines. The detected translated transcript and transcript fractions were consistent with previously reported data: a coverage of from 60% to 70% (depending on the mRNA extraction protocol) of PCGs with translated transcripts was shown in the HEK293 embryonic kidney cell line at an RPKM cutoff of >0.5 [23]. For transcriptome data of healthy human liver tissue, the transcriptome coverage in repeats was up to 95% at an RPKM threshold of >0 [24]. Table 2 presents the number of PCGs for which transcripts were detected by RNA-seq (a), translated transcripts by Ribo-seq (b), translated transcripts by RNC-seq (c), and detected proteins by LC-MS/MS (d).

The fraction of PCGs with detected mRNA translation was quite high and amounted to about 80%, on average, regardless of the experimental method used (RNC-seq or Ribo-seq). Modern experimental methods detected at least one transcript and translated transcript with a non-zero expression level for 80% of protein-coding genes. By comparison, at the proteomic level (Table 2), panoramic mass spectrometry detected a protein product for only 30% of protein-coding genes.

**Figure 2:**
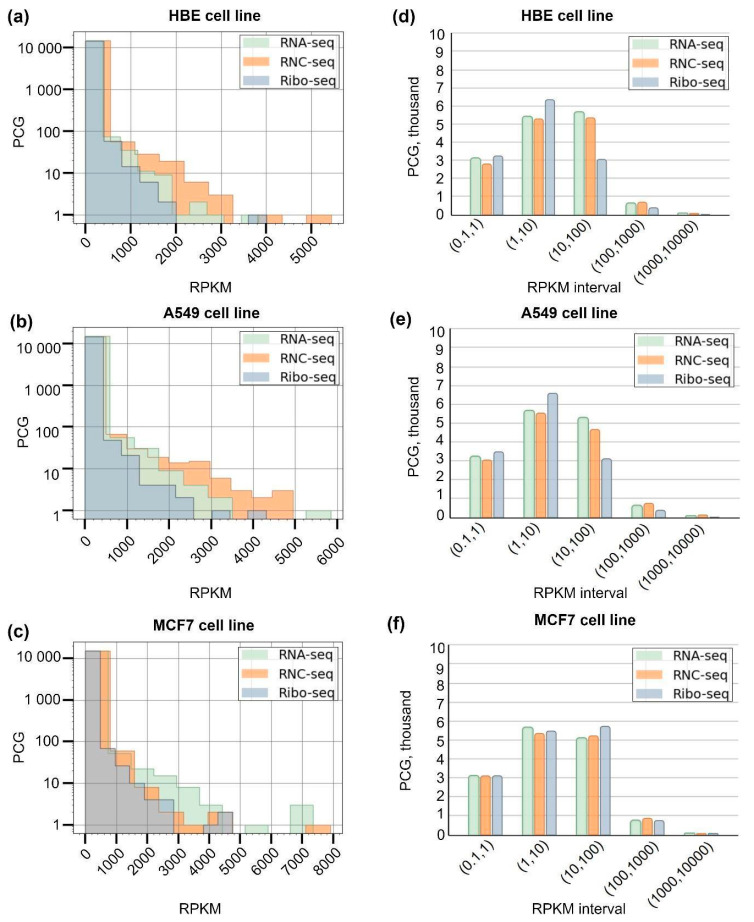
Distribution histograms (**a**–**c**) and interval histograms for transcripts (RNA-seq) (**d**) and translated transcripts (Ribo-seq and RNC-seq) (**e**,**f**) detected in HBE, A549, and MCF-7 cell lines in RPKM expression intervals (0.1, 1), (1, 10), (100, 1000), and (1000, 10,000). The Y-scale in histograms (**a**–**c**) is shown as a log10 scale.

The distribution pattern of the identified transcripts and translated transcripts, depending on the RPKM value, is shown in Figure 2. The analyzed cell lines were characterized by a decrease in the number of detected transcripts and translated transcripts with an increase in RPKM (histograms (a–b)). Most transcripts and translated transcripts were characterized by RPKM values of <10. The number of transcripts and translated transcripts detected at an RPKM of >500 was low and amounted to several tens in each case (Figure 2a–c).

The distribution pattern of transcripts (RNA-seq) repeats that of translated transcripts (RNC and Ribo-seq): a peak in the range between 0.1 and 1 reflects the PCG fraction (about 3000 transcripts and translated transcripts) with low RPKM values (Figure 2d–f). Approximately 6800 PCG translated transcripts were detected in the 1–10 RPKM interval by Ribo-seq in the HBE and A549 cell lines.

The largest number of PCG translated transcripts was detected in the 1–10 interval by Ribo-seq (HBE and A549 cell lines), while for the MCF-7 cell line, both translatome methods revealed comparable numbers of PCGs in all intervals. The number of genes detected in high-copy intervals (100, 1000) and (1000, 10,000) in the transcriptome and translatome was comparable with each other and was not more than 1000 genes for each cell line.

Genes encoding housekeeping proteins—ACTB (actin beta), GAPDH (glyceraldehyde-3-phosphate dehydrogenase), and EEF1A1 (eukaryotic translation elongation factor 1 alpha 1)—displayed high expression at the mRNA, translatome, and proteome levels. For example, a transcript corresponding to the translation elongation factor (EEF1A1) was detected in the HBE cell line at an RPKM level of 3829. The corresponding translated transcript was detected at a higher value (RPKM = 5437) in the same sample analyzed by RNC-seq. The opposite situation was observed when the RPKM level of a transcript was higher than that of the corresponding translated transcript for the ribosomal protein L41 (RPL41): the RPKM was 7354 for mRNA, and the RPKM was 4948 for the translated transcript detected by RNC-seq. Despite the lack of a direct quantitative correlation between the RPKM signal for mRNA and the amount of the corresponding protein, there was a trend that PCGs, whose transcripts and translated transcripts were detected at a relatively higher RPKM level, were more often detected at the proteome level: proteins encoded by the GAPDH and EEF1A1 genes were detected in the A549 and MCF-7 cell lines at an LFQ intensity value of 1975 and 3633, respectively.

### 2.3. Translatome Profiling Methods Reveals Highly Comparable Sets of Genes

The concordance of the translatome profiles obtained by the two different methods (Ribo-seq and RNC-seq) was assessed using interval calculations of the Tanimoto coefficient [25]. These calculations enable assessing the similarity of two datasets (in this case, the lists of translated transcripts detected by each method at an RPKM value above the threshold). The higher the Tanimoto coefficient, the more matches between the lists.

**Figure 3:**
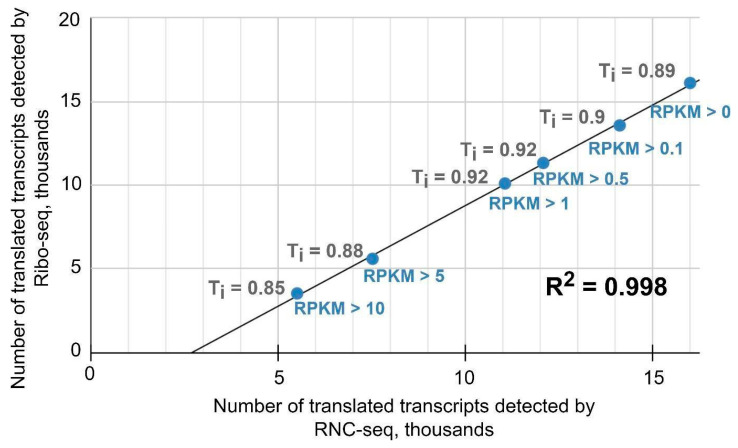
Tanimoto coefficient values obtained by comparing translated transcript sets detected by Ribo-seq and RNC-seq at different RPKM threshold values in the MCF-7 cell line.

As an example, Figure 3 shows a plot of the interval values for the translatome profiles in the MCF-7 cell line. It can be seen that comparison of translated transcript sets at an RPKM of >0 gave a Tanimoto coefficient of 0.89, i.e., the lists were almost 90% similar. As the cutoff level increased, the Tanimoto coefficient decreased slightly, reaching a value of 0.85 at an RPKM of >10. There was a high degree of concordance between quantitative data―the RPKM value for translated transcripts detected by Ribo-seq and that for translated transcripts detected by RNC-seq. By comparison, the Tanimoto coefficient values at an RPKM of >0 were similar at 0.86 for HBE and 0.87 for A549.

We also found that the correlation between the log10 (RPKM) value of translated transcripts detected by Ribo-seq and those detected by RNC-seq (total number of translated transcripts n = 15,676) in the MCF-7 cell line was R^2^ = 0.96. Similar indicators were obtained for the HBE and A549 cell lines, at 0.87 and 0.85, respectively. We also plotted the correlation of the RPKM between the two translatome methods (Figure S1). All cell lines showed a *p*-value < 0.001.

**Figure 4:**
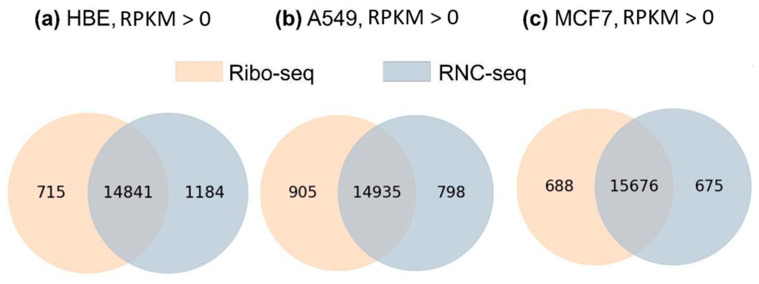
Venn diagrams showing intersections of PCG lists with translated transcripts detected by RNC-seq and Ribo-seq (RPKM > 0) in (**a**) HBE cells (n = 16,740), (**b**) A549 cells (n = 16,638), and (**c**) MCF-7 cells (n = 17,039).

The obtained results show that despite the fundamental difference in the basics of the methodologies in translatome analysis (Ribo-seq analyzes short ribosome-protected fragments, and RNC-seq analyzes ribosome-associated mRNAs), the PCG fraction with the detected translated transcripts was comparable. In addition, the comparability of transatomic profiling methods shows high reproducibility among several Ribo-seq and RNC-seq experiments performed on several samples of MCF7 cell line (Figure S2). Both methods provided information about the level of at least one translated transcript for each of 80% of human PCGs, and the results of quantitative measurements of a translation level correlated between the methods (R^2^ = 0.892). In Figure 4, Venn diagrams demonstrate intersections of the translated transcript lists detected by each method (Ribo-seq and RNC-seq, RPKM > 0). It can be seen that the PCG fraction with translated transcripts detected by both methods was quite high and ranged from 88% (HBE) to 92% (MCF-7). The number of translated transcripts detected by only one of the methods was also comparable for the A549 and MCF-7 cell lines and accounted for only 4–6% of the translated transcripts detected by both methods. Despite the fact that the total number of translated transcripts was comparable in both methods, there was a translatome fraction that was detected by only one of the methods.

We obtained lists of uniquely detected transcripts for the MCF7 cell line using the Ribo-seq and RNC-seq methodologies, identifying a total of 688 and 675 transcripts, respectively (Figure 4c). These transcript lists were subsequently annotated based on key parameters, including transcript length (considering only canonical transcripts) and GC content percentage (Table S2). This detailed annotation allowed for a comprehensive comparison of the characteristics of the detected transcripts, providing insights into the transcriptional landscape of the MCF7 cell line, as revealed by these advanced sequencing techniques.

The analysis of the GC content within the uniquely identified transcripts revealed a comparable distribution between the two sequencing methodologies employed. Specifically, the median GC content for transcripts detected via RNC-seq was found to be 46%, while for those identified through Ribo-seq, it was slightly lower at 45.5%. This similarity in the GC content suggests a consistent transcriptional profile across both techniques, reflecting the inherent characteristics of the MCF7 cell line.

Moreover, we assessed the average lengths of the detected mRNA transcripts, which were determined to be 2737 nucleotides for RNC-seq and 2529 nucleotides for Ribo-seq. It is noteworthy that through the application of ribosome profiling (Ribo-seq), a gene with an unprecedented length of 30,609 base pairs was identified, specifically, the GRIN2B gene, which encodes for the glutamate ionotropic receptor NMDA type subunit 2B. In contrast, the longest transcript detected via RNC sequencing (RNC-seq) measured 20,374 base pairs and corresponded to the NKAIN3 gene, which is associated with sodium/potassium transporting ATPase interacting 3. This disparity in transcript lengths between Ribo-seq and RNC-seq highlights the unique capabilities of ribosome profiling in capturing full-length coding sequences, thereby providing deeper insights into gene expression and translational dynamics.

### 2.4. Gene-Centric Molecular “Portrait” Shows High Concordance between the Transcriptome and the Translatome

We performed comparative gene-centric analyses of the lists of detected transcripts, translated transcripts, and proteins in the A549 and MCF-7 cell lines. The results are presented in Table 3. The Tanimoto coefficient between the lists was calculated for each cell line. The list of detected transcripts and translated transcripts was formed at different RPKM cutoff levels.

Regardless of the method, the translatome profile of the analyzed cell lines largely (>85%) repeated the results of the transcriptome analysis in the same sample. According to the results of the translatome analysis, almost all the detected transcripts in the same sample were characterized by the presence of the corresponding mRNA, i.e., mRNA in the ribosomal complex.

The results of translatome analysis show the presence of translating mRNAs [26]. We suggest that translation results in the corresponding proteins. The obtained results indicate that proteins (detected by LC-MS/MS) in the same samples were found only for 30–40% of the translated transcripts. The calculated Tanimoto coefficients did not alter the observed phenomenon as the RPKM threshold value increased. The translated transcript lists (RNC-seq) were slightly closer to the transcriptome profile than the translated transcript list (Ribo-seq), which was slightly more similar to the proteome profile compared with RNC-seq.

**Figure 5:**
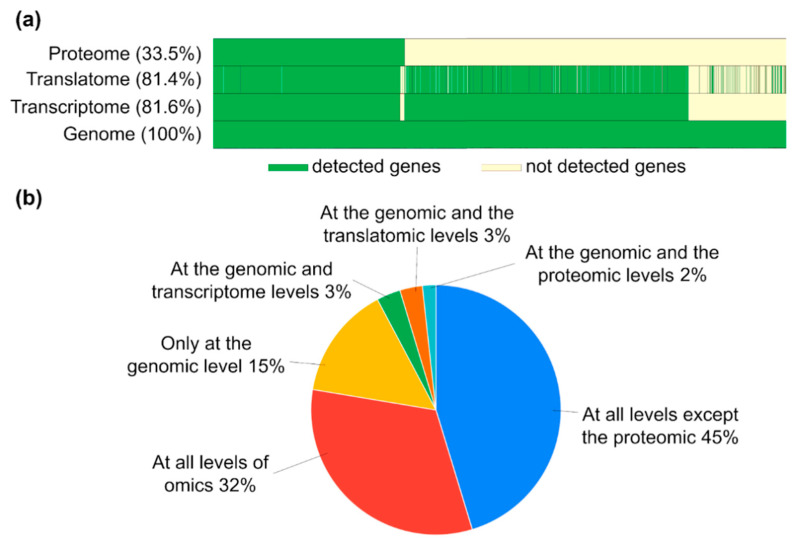
Gene-centric visualization of the results of multiomic analysis of protein-coding genes in the MCF-7 cell line, with (**a**) a molecular “portrait” of the biological sample, which includes information on transcriptome, translatome, and proteome levels of implementation of genome-encoded information. Genome coverage percentage of detected transcripts and translated transcripts (RNC-seq) at RPKM > 0 and proteins in proteomic LC-MS/MS analysis at an LFQ intensity of >0 is indicated. (**b**) Transcripts, translated transcripts, and protein fractions of the total PCGs (20,423 genes), which were detected at different omics levels.

Figure 5 summarizes the results of the multiomic analysis of the MCF-7 cell line, characterized by the highest correlation between the translatome methods and the Tanimoto similarity coefficients reported in this paper.

The resulting molecular “portrait” of the MCF-7 cell line showed that transcriptome and translatome omics levels covered an equal PCG percentage of their total number, with 81.6% and 81.4%, respectively (Figure 5a). Thus, we may suggest that most mRNAs expressed in MCF-7 are involved in the translation process; however, only 33.5% of the proteins could be detected at the proteome level. This led to a high PCG fraction of 45% (9330 PCGs) detected at all omics levels, except proteome one (Figure 5b). Also, about 15% of the genes existed only at the genome level and did not express products at other omics levels.

A similar molecular “portrait” and similar coverage patterns of transcriptome, translatome, and proteome profiles were observed in the A549 cell line, which proves that this loss of information caused by the transition from different omics levels was associated with the features of these experimental technologies.

The proposed method for visualizing a gene-centric molecular “portrait” of a biological sample, which includes information on the transcriptome, translatome, and proteome levels of genome-encoded information implementation, reveals molecular events on a gene-centric scale. Nevertheless, transcriptome and translatome profiling describes biological processes with allowance for individual splice forms.

## 3. Discussion

The central dogma of molecular biology [27] in combination with the results of multiomic studies hypothesizes the need to estimate the ratio of the numbers of protein-coding genes and their corresponding mRNAs and proteins. Presumably, the number of transcribed mRNA regions is 20% higher than that of translated ones, and the number of protein products is, on average, 10% less than that of translated mRNA regions [28]. This is confirmed by studies of various species (human, macaque, mouse, opossum, chicken, and platypus) [1].

The number of transcribed and translated mRNA regions can be the same. The conducted analysis shows that the difference can be only 3% of the PCG number, regardless of the chosen translatome analysis method (Ribo-seq or RNC-seq). Almost identical transcript and translated transcript lists can result from the features of translation regulation or an object of study (cell line).

The total amount of ribosomes in mammalian cells is closely related to the rate of their proliferation. In actively dividing cells, ribosomal RNAs (rRNAs) account for about 80% of all nucleic acids and about 15% of the biomass [29]. Expecting that all translation- initiating ribosomes complete this process and that polypeptide chains elongate at the same rate along all transcripts, the ribosomal footprint density is usually used to assess the efficiency of translation. This parameter is calculated as the number of protein molecules produced per mRNA molecule per unit time [30]. Translation occurs rapidly, at a rate of ~20 amino acids per second in vivo [31,32], which is equivalent to 50 ms per codon [33]. It has been demonstrated [34] that the translation elongation rate varies in different human organs and depends on the metabolic rate. Differences can amount to over 50%, with the highest and lowest translation rates being observed in the liver and skeletal muscle, respectively.

The experimental methods of translatome analysis evaluated in this study do not account for the stage of the process, namely, initiation, elongation, or termination. A study in *E. coli* [35] demonstrated that 90% of both ribosome subunits are involved in translation at any particular time point, and the 30S and 50S ribosomal subunits spend the same average time bound to an mRNA. Comparing estimates of the average mRNA bound time and the time required for the translation of an average protein, the authors suggest that ≥50% of 70S complexes re-initiate in translation. There are no similar studies in cell lines, so it is difficult to assess the variability in the translation rate under different external conditions.

While this study primarily focuses on translational factors contributing to transcriptome-translatome discordance, it is important to acknowledge that proteomic limitations also play a significant role in the discrepancy. Several technical factors inherent to proteomics, such as single-enzyme digestion protocols, can lead to incomplete proteome maps. For example, tryptic digestion, which was used in the proteomic datasets referenced in this study [36], often provides limited coverage due to its inability to fully digest certain protein regions. As noted in recent studies, including that by Sinitsyn et al. [37], the use of multiple proteolytic enzymes can significantly improve proteome coverage. Although multi-enzyme digestion protocols have not yet been applied to the cell lines used in our study, future research in this direction could greatly improve the completeness of proteome maps and reduce the transcriptome–proteome discordance observed in such datasets.

The translation elongation rate varies up to a factor of ~20 in different PCGs and significantly correlates with the translation initiation rate [30]. The translation elongation rate is impacted by the amino acid composition of synthesized proteins as much as codon and transfer RNA (tRNA) adaptation. Elongation occurs slowly along transcripts encoding ribosomal proteins, which have a lower protein yield compared with other transcripts with a similar ribosome density. The side-chain size and charge of the incorporated amino acid affect the rate of polypeptide chain elongation, as do cotranslational protein folding and interaction with chaperones [38]. Negatively charged proteins are synthesized about 2-fold faster, on average, than positively charged proteins. The amino acid charge and relative abundance of cognate tRNAs impact the translation elongation rate to a similar degree.

We suggest that the experimental translatomic approaches, RNC-seq and Ribo-seq, should be combined with functional analysis and in vivo approaches to measuring the translation elongation rate. Transcripts are transient messengers that often degrade, while proteins can persist for a longer time, depending on their stability and the needs of cells. If the difference between analytical methods in nucleic acid sequencing and proteome research is neglected, the lack of linear relationships between the number of transcripts and a protein level may be explained by complex regulatory mechanisms, such as alternative splicing and translational control. A full understanding of mRNA translation and its regulation is possible only via measuring the kinetics of mRNA translation in a single cell.

## 4. Materials and Methods

Initial data were downloaded from the TranslatomeDB database [22]. The availability of experiments on the same biological material using the RNA-seq, RNC-seq, and Ribo-seq methods was used as the dataset selection criterion. Thus, the sequencing data of three cell lines of different origin were selected: HBE (normal human bronchial epithelial cells), A549 (human lung adenocarcinoma epithelial cells), and MCF-7 (hormone-responsive breast cancer cells). Source and sequencing platform details are listed in Table 4.

Cell lines with comparable sequencing depth and sample preparation were chosen as control samples [41]. All datasets were acquired using an Illumina sequencing platform with a cDNA sequencing library. The data were processed by a single pipeline incorporated to TranslatomeDB, which included alignment by the FANSe3 algorithm to the reference genome and quantification by the RSEM program [22]. The data were aligned to the reference genome version GRCh37 (hg19, RefSeq GCF_000001405.13).

For transcriptome (RNA-seq) and translatome (RNC-seq and Ribo-seq) analyses, one dataset for each cell line was selected; a total of nine datasets were selected (see Table 4).

Mass spectrometry data for the proteome of the A549 and MCF-7 cell lines were obtained from the NCI60 dataset (46 cell lines and 8000 genes) [36]. This study used semi-quantitative LC-MS/MS analysis of 8000 protein-coding genes, whose corresponding protein products were detected in at least 1 of the 46 selected cell lines.

For comparative multiomic (transcriptomic, translatomic, and proteomic) analysis, we used a gene-centric approach [24]. Information about splice variants was pooled, and each protein-coding gene was associated with a transcript or translated transcript expression value expressed in RPKM units, which is a convenient measure for comparing different methods within a sample [42]. In the proteome data, protein abundance is presented as the label-free quantification (LFQ) intensity [43].

The initial datasets were represented by a set of 26,000 different genes. In our analysis, gene-centric data of 20,423 protein-coding genes from the UniProt database (Release 2023_03) with the “reviewed” status were used. An RPKM threshold value of >0 was chosen as the selected transcript and translated transcript expression level [24] (Table S1).

To identify fractions of low-, medium-, and high-copy genes [25], we used the following RPKM intervals: low-copy (0.1, 1), medium-copy (1, 10), (10, 100), and high-copy (100, 1000), (1000, 10,000).

The choice of RPKM levels was based on the need to balance sensitivity and specificity in detecting transcripts [24]. This was crucial for filtering out lowly expressed genes and focusing on genes with significant expression levels. In a previous study, transcripts of genes with low RPKM values (0 < RPKM < 1) were detected by PCR; thus, cut-off higher than 0 (RPKM > 0) could be applied for the analysis. By using different RPKM levels, we were able to assess the transcriptome coverage and gene expression patterns in the datasets.

Thus, we used an approach based on comparison of the number of translated transcripts and transcripts expressed at different thresholds in all samples [44]. We used thresholds of 0 [45], 0.1 [46], 1 [47], 5 [48], and 10 [49], which promoted the determination of a threshold for expression above noise and accounted for many factors, in particular, the sequencing depth and technical variability.

To calculate the semantic similarity *T*(*a*, *b*) of the sets of transcripts a and b, we used the Tanimoto normalization equation [50]:

$$T(a,\;b)=\frac{\left(P|ab|\right)}{\left(|Pa|+|Pb|-|Pab|\right)},$$

where *Pa* is the diversity of the set a transcripts; *Pb* is the diversity of the set b transcripts; and *Pab* is the diversity of the shared *a* and *b* transcripts. A Tanimoto coefficient *T*(*a*, *b*) of between 1.0 and 0.7, 0.7 and 0.55, or less than 0.55 means that the two sets are identical, weakly similar, or significantly different, respectively [25].

The Tanimoto coefficient was used to compare the results of the gene-centric transcriptome, translatome, and proteome profiling of the same sample. For all Spearman correlation coefficients, *p*-values were estimated using exact permutations tests.

## 5. Conclusions

The translatome methods, Ribo-seq and RNC-seq, detected approximately 80% of human protein-coding genes, which is an accurate characterization of genome-wide translation. The distribution pattern of the number of transcripts and translated transcripts detected at targeted RPKM cutoffs was comparable for the RNA-seq, RNC-seq, and Ribo-seq results. A high degree of similarity (the Tanimoto coefficient) and a high correlation between the RPKM values for the translated transcripts detected by RNC-seq and Ribo-seq were shown. Both translatome profiling methods detected at least one translated transcript for each of the 90% PCGs. Such results may be a consequence of the use of the ultra-sensitive FANSe3 alignment algorithm, so the question of the list of programs for processing translatome sequencing data still remains open. When selecting the most optimal software, it will be possible to distinguish between a translatome and a transcriptome of cells and cell lines. However, RNC-sec is characterized by a simpler protocol for preparing samples and libraries for sequencing, which makes it the method of choice for survey studies of the translatome.

Translatome analysis effectively reflects the phenotype of cell lines and their functional differences. Despite different operating procedures, RNC-seq and Ribo-seq methods provide similar results in terms of the number of sequenced translated transcripts. Unlike RNC-seq, Ribo-seq is more complex to perform (Figure S3); it requires specialized laboratory procedures, such as cell processing, ribosome fragmentation, and subsequent sequencing, but overall, this study emphasizes that it does not really matter which method is used to evaluate the translatome.

## Figures and Tables

**Table 1:** Results of queries for ‘Ribo-seq’ and ‘RNC-seq’ experiments in databases.

Database	Ribo-Seq ^1^	RNC-Seq ^2^
	Number of entries	
Pubmed	1454	210
PMC Full-Text	8265	1278
BioProject	1339	27
GEO DataSets	8168	36
DB Translatome	4054	216

^1^—“ribosome-profiling” OR “riboseq” OR “ribo-seq” OR “ribosome profiling” OR “ribosome footprint” OR “ribosome footprinting” OR “ribosome-footprinting”; ^2^—“RNC-seq” OR “ribosome-nascent chain” OR “Ribosome nascent-chain complex-bound mRNA sequencing”.

**Table 2:** Number of protein-coding genes (PCGs, Uniprot Release 2023_03, a total of 20,423 PCGs in the human exome) in each cell line (HBE, A549, MCF-7), for which products at transcriptome (RPKM > 0) and translatome (RPKM > 0) levels and proteins were detected.

Level	Method	Number of PCGs (% of the Total PCGs)		
		HBE (Normal Human Bronchial Epithelial Cells)	A549 (Lung Adenocarcinoma Epithelial Cells)	MCF-7 (Hormone- Responsive Breast Cancer Cell Line)
Transcriptome	RNA-seq	16,375 (80.2%)	16,807 (82.3%)	16,655 (81.6%)
Translatome	RNC-seq	16,300 (79.8%)	15,999 (78.3%)	16,622 (81.4%)
	Ribo-seq	15,815 (77.4%)	16,112 (78.9%)	16,641 (81.5%)
Proteome	LC-MS/MS	–	5850 (28.6%)	6843 (33.5%)

**Table 3:** Tanimoto’s similarity coefficient between translatome, transcriptome, and proteome data at different RPKM cutoffs (RPKM > 0, RPKM > 1, RPKM > 10) calculated for (a) HBE, (b) A549, and (c) MCF-7 cell lines. For the proteome data, an LFQ intensity cutoff higher than 0 was applied.

(a) HBE			
	RPKM > 0	RPKM > 1	RPKM > 10
RNA-seq vs. RNC-seq	0.91	0.95	0.87
RNA-seq vs. Ribo-seq	0.89	0.83	0.53
**(b) A549**			
	**RPKM > 0**	**RPKM > 1**	**RPKM > 10**
RNA-seq vs. RNC-seq	0.91	0.93	0.81
RNA-seq vs. Ribo-seq	0.9	0.86	0.57
Proteome vs. RNC-seq	0.36	0.32	0.17
Proteome vs. Ribo-seq	0.35	0.34	0.19
**(c) MCF-7**			
	**RPKM > 0**	**RPKM > 1**	**RPKM > 10**
RNA-seq vs. RNC-seq	0.92	0.97	0.93
RNA-seq vs. Ribo-seq	0.92	0.95	0.86
Proteome vs. RNC-seq	0.4	0.32	0.17
Proteome vs. Ribo-seq	0.4	0.31	0.17

**Table 4:** Initial sequencing data obtained by transcriptomic and translatomic methods for three cell lines (HBE, A549, MCF-7).

Cell Line	Assay ^1^	Number of Reads × 10^6^	Sequence Read Archive (SRA)	Study Reference	Illumina Sequencer Model
**HBE** (normal human bronchial epithelial cells)	RNA-seq	12	SRR611121	[14]	Genome Analyzer IIx
	RNC-seq	16	SRR611122		
	Ribo-seq	110	SRR3286543	[39]	HiSeq 2500
**A549** (lung adenocarcinoma epithelial cells)	RNA-seq	19	SRR611119	[14]	Genome Analyzer IIx
	RNC-seq	13	SRR611120		
	Ribo-seq	95	SRR3286544	[39]	HiSeq 2500
**MCF-7** (hormone—responsive breast cancer cell line)	RNA-seq	19	SRR6892923	[40]	HiSeq 2000
	RNC-seq	17	SRR6892909		
	Ribo-seq	13	SRR6892903		

^1^ RNA-seq (high-throughput mRNA sequencing) and translatomic methods: (1) RNC-seq (ribosome–nascent chain complex-bound mRNA sequencing); (2) Ribo-seq (ribosome profiling, sequencing of ribosome-protected mRNA fragments).

## Data Availability

Data are contained within the article and Supplementary Materials.

## Supplementary Materials

The following supporting information can be downloaded at: https://www.mdpi.com/article/10.3390/ijms252010970/s1.

## References

[1] Chang C., Li L., Zhang C., Wu S., Guo K., Zi J., Chen Z., Jiang J., Ma J., Yu Q. (2014). Systematic Analyses of the Transcriptome, Translatome, and Proteome Provide a Global View and Potential Strategy for the C-HPP. J. Proteome Res..

[2] Gawron D., Gevaert K., Van Damme P. (2014). The Proteome under Translational Control. Proteomics.

[3] Qanmber G., You Q., Yang Z., Fan L., Zhang Z., Chai M., Gao B., Li F., Yang Z. (2023). Transcriptional and Translational Landscape Fine-Tune Genome Annotation and Explores Translation Control in Cotton. J. Adv. Res..

[4] Neuhaus K., Landstorfer R., Fellner L., Simon S., Schafferhans A., Goldberg T., Marx H., Ozoline O.N., Rost B., Kuster B. (2016). Translatomics Combined with Transcriptomics and Proteomics Reveals Novel Functional, Recently Evolved Orphan Genes in *Escherichia coli* O157:H7 (EHEC). BMC Genom..

[5] Rendleman J., Cheng Z., Maity S., Kastelic N., Munschauer M., Allgoewer K., Teo G., Zhang Y.B.M., Lei A., Parker B. (2018). New Insights into the Cellular Temporal Response to Proteostatic Stress. eLife.

[6] Kitchen R.R., Rozowsky J.S., Gerstein M.B., Nairn A.C. (2014). Decoding Neuroproteomics: Integrating the Genome, Translatome and Functional Anatomy. Nat. Neurosci..

[7] Anderson L., Seilhamer J. (1997). A comparison of selected mRNA and protein abundances in human liver. Electrophoresis.

[8] Gygi S.P., Rochon Y., Franza B.R., Aebersold R. (1999). Correlation between protein and mRNA abundance in yeast. Mol. Cell Biol..

[9] Ghazalpour A., Bennett B., Petyuk V.A., Orozco L., Hagopian R., Mungrue I.N., Farber C.R., Sinsheimer J., Kang H.M., Furlotte N. (2011). Comparative analysis of proteome and transcriptome variation in mouse. PLoS Genet..

[10] Yan X., Hoek T.A., Vale R.D., Tanenbaum M.E. (2016). Dynamics of Translation of Single mRNA Molecules In Vivo. Cell.

[11] Wunner W.H., Bell J., Munro H.N. (1966). The Effect of Feeding with a Tryptophan-Free Amino Acid Mixture on Rat-Liver Polysomes and Ribosomal Ribonucleic Acid. Biochem. J..

[12] Mašek T., Valášek L., Pospíšek M., Nielsen H. (2011). Polysome Analysis and RNA Purification from Sucrose Gradients. RNA.

[13] Spangenberg L., Shigunov P., Abud A.P.R., Cofré A.R., Stimamiglio M.A., Kuligovski C., Zych J., Schittini A.V., Costa A.D.T., Rebelatto C.K. (2013). Polysome Profiling Shows Extensive Posttranscriptional Regulation during Human Adipocyte Stem Cell Differentiation into Adipocytes. Stem Cell Res..

[14] Wang T., Cui Y., Jin J., Guo J., Wang G., Yin X., He Q.-Y., Zhang G. (2013). Translating mRNAs Strongly Correlate to Proteins in a Multivariate Manner and Their Translation Ratios Are Phenotype Specific. Nucleic Acids Res..

[15] Ingolia N.T., Brar G.A., Rouskin S., McGeachy A.M., Weissman J.S. (2012). The Ribosome Profiling Strategy for Monitoring Translation in Vivo by Deep Sequencing of Ribosome-Protected mRNA Fragments. Nat. Protoc..

[16] McGlincy N.J., Ingolia N.T. (2017). Transcriptome-Wide Measurement of Translation by Ribosome Profiling. Methods.

[17] Ingolia N.T., Hussmann J.A., Weissman J.S. (2019). Ribosome Profiling: Global Views of Translation. Cold Spring Harb. Perspect. Biol..

[18] Shirokikh N.E., Archer S.K., Beilharz T.H., Powell D., Preiss T. (2017). Translation Complex Profile Sequencing to Study the in Vivo Dynamics of mRNA–Ribosome Interactions during Translation Initiation, Elongation and Termination. Nat. Protoc..

[19] Heiman M., Kulicke R., Fenster R.J., Greengard P., Heintz N. (2014). Cell Type-Specific mRNA Purification by Translating Ribosome Affinity Purification (TRAP). Nat. Protoc..

[20] Hafner M., Katsantoni M., Köster T., Marks J., Mukherjee J., Staiger D., Ule J., Zavolan M. (2021). CLIP and Complementary Methods. Nat. Rev. Methods Primers.

[21] Tarbeeva S., Lyamtseva E., Lisitsa A., Kozlova A., Ponomarenko E., Ilgisonis E. (2021). ScanBious: Survey for Obesity Genes Using PubMed Abstracts and DisGeNET. J. Pers. Med..

[22] Liu W., Xiang L., Zheng T., Jin J., Zhang G. (2018). TranslatomeDB: A Comprehensive Database and Cloud-Based Analysis Platform for Translatome Sequencing Data. Nucleic Acids Res..

[23] Sultan M., Amstislavskiy V., Risch T., Schuette M., Dökel S., Ralser M., Balzereit D., Lehrach H., Yaspo M.-L. (2014). Influence of RNA Extraction Methods and Library Selection Schemes on RNA-Seq Data. BMC Genom..

[24] Ilgisonis E.V., Ponomarenko E.A., Tarbeeva S.N., Lisitsa A.V., Zgoda V.G., Radko S.P., Archakov A.I. (2022). Gene-Centric Coverage of the Human Liver Transcriptome: QPCR, Illumina, and Oxford Nanopore RNA-Seq. Front. Mol. Biosci..

[25] Ilgisonis E., Vavilov N., Ponomarenko E., Lisitsa A., Poverennaya E., Zgoda V., Radko S., Archakov A. (2021). Genome of the Single Human Chromosome 18 as a “Gold Standard” for Its Transcriptome. Front. Genet..

[26] Zhang S., Chen Y., Wang Y., Zhang P., Chen G., Zhou Y. (2020). Insights Into Translatomics in the Nervous System. Front. Genet..

[27] Crick F. (1970). Central Dogma of Molecular Biology. Nature.

[28] Wang Z.-Y., Leushkin E., Liechti A., Ovchinnikova S., Mößinger K., Brüning T., Rummel C., Grützner F., Cardoso-Moreira M., Janich P. (2020). Transcriptome and Translatome Co-Evolution in Mammals. Nature.

[29] Lane A.N., Fan T.W.-M. (2015). Regulation of Mammalian Nucleotide Metabolism and Biosynthesis. Nucleic Acids Res..

[30] Riba A., Di Nanni N., Mittal N., Arhné E., Schmidt A., Zavolan M. (2019). Protein Synthesis Rates and Ribosome Occupancies Reveal Determinants of Translation Elongation Rates. Proc. Natl. Acad. Sci. USA.

[31] Sørensen M.A., Pedersen S. (1991). Absolute in Vivo Translation Rates of Individual Codons in *Escherichia coli*. J. Mol. Biol..

[32] Zhu M., Dai X., Wang Y.-P. (2016). Real Time Determination of Bacterial in Vivo Ribosome Translation Elongation Speed Based on LacZα Complementation System. Nucleic Acids Res..

[33] Prabhakar A., Choi J., Wang J., Petrov A., Puglisi J.D. (2017). Dynamic Basis of Fidelity and Speed in Translation: Coordinated Multistep Mechanisms of Elongation and Termination. Protein Sci..

[34] Gerashchenko M.V., Peterfi Z., Yim S.H., Gladyshev V.N. (2021). Translation Elongation Rate Varies among Organs and Decreases with Age. Nucleic Acids Res..

[35] Metelev M., Lundin E., Volkov I.L., Gynnå A.H., Elf J., Johansson M. (2022). Direct Measurements of mRNA Translation Kinetics in Living Cells. Nat. Commun..

[36] Gholami A.M., Hahne H., Wu Z., Auer F.J., Meng C., Wilhelm M., Kuster B. (2013). Global Proteome Analysis of the NCI-60 Cell Line Panel. Cell Rep..

[37] Sinitcyn P., Richards A.L., Weatheritt R.J., Brademan D.R., Marx H., Shishkova E., Meyer J.G., Hebert A.S., Westphall M.S., Blencowe B.J. (2023). Global detection of human variants and isoforms by deep proteome sequencing. Nat. Biotechnol..

[38] Lu J., Hua Z., Kobertz W.R., Deutsch C. (2011). Nascent Peptide Side Chains Induce Rearrangements in Distinct Locations of the Ribosomal Tunnel. J. Mol. Biol..

[39] Lu S., Zhang J., Lian X., Sun L., Meng K., Chen Y., Sun Z., Yin X., Li Y., Zhao J. (2019). A Hidden Human Proteome Encoded by ‘Non-Coding’ Genes. Nucleic Acids Res..

[40] Clamer M., Tebaldi T., Lauria F., Bernabò P., Gómez-Biagi R.F., Marchioretto M., Kandala D.T., Minati L., Perenthaler E., Gubert D. (2018). Active Ribosome Profiling with RiboLace. Cell Rep..

[41] Pyatnitskiy M.A., Arzumanian V.A., Radko S.P., Ptitsyn K.G., Vakhrushev I.V., Poverennaya E.V., Ponomarenko E.A. (2021). Oxford Nanopore MinION Direct RNA-Seq for Systems Biology. Biology.

[42] Zhao S., Ye Z., Stanton R. (2020). Misuse of RPKM or TPM Normalization When Comparing across Samples and Sequencing Protocols. RNA.

[43] Luber C.A., Cox J., Lauterbach H., Fancke B., Selbach M., Tschopp J., Akira S., Wiegand M., Hochrein H., O’Keeffe M. (2010). Quantitative Proteomics Reveals Subset-Specific Viral Recognition in Dendritic Cells. Immunity.

[44] Koch C.M., Chiu S.F., Akbarpour M., Bharat A., Ridge K.M., Bartom E.T., Winter D.R. (2018). A Beginner’s Guide to Analysis of RNA Sequencing Data. Am. J. Respir. Cell Mol. Biol..

[45] Dall’Agnol H.P., Baraúna R.A., De Sá P.H., Ramos R.T., Nóbrega F., Nunes C.I., Das Graças D.A., Carneiro A.R., Santos D.M., Pimenta A.M. (2014). Omics Profiles Used to Evaluate the Gene Expression of Exiguobacterium Antarcticum B7 during Cold Adaptation. BMC Genom..

[46] Abdullah H.M., Akbari P., Paulose B., Schnell D., Qi W., Park Y., Pareek A., Dhankher O.P. (2016). Transcriptome Profiling of Camelina Sativa to Identify Genes Involved in Triacylglycerol Biosynthesis and Accumulation in the Developing Seeds. Biotechnol. Biofuels.

[47] Łabaj P.P., Kreil D.P. (2016). Sensitivity, Specificity, and Reproducibility of RNA-Seq Differential Expression Calls. Biol. Direct.

[48] Yang J.-R., Chen X. (2019). Dosage Sensitivity of X-Linked Genes in Human Embryonic Single Cells. BMC Genom..

[49] Wright H.L., Thomas H.B., Moots R.J., Edwards S.W. (2013). RNA-Seq Reveals Activation of Both Common and Cytokine-Specific Pathways Following Neutrophil Priming. PLoS ONE.

[50] Rogers D.J., Tanimoto T.T. (1960). A Computer Program for Classifying Plants: The Computer Is Programmed to Simulate the Taxonomic Process of Comparing Each Case with Every Other Case. Science.

