# Supplementary material for: The Translatome Map: RNC-Seq vs. Ribo-Seq for Profiling of HBE, A549, and MCF-7 Cell Lines
Source: Int J Mol Sci. 2024 Oct 12;25(20):10970. doi: 10.3390/ijms252010970 (PMC11507076; doi:10.3390/ijms252010970)
Supplement: Supplementary file 1 [file ijms-25-10970-s001.zip › Figure S1-S3.pdf]

The Translatome Map: RNC-Seq vs Ribo-Seq for Profiling of HBE, A549, and MCF- 7 Cell Lines

Supplementary Materials

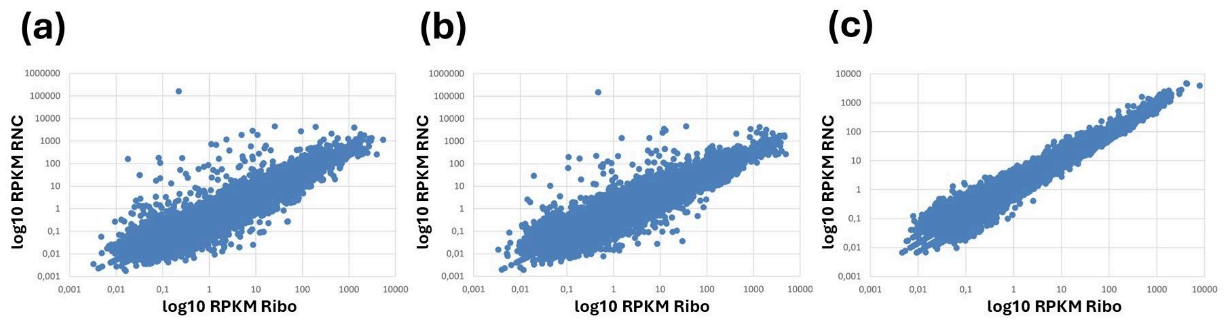

Figure S1. Correlation plot of RPKM expression values for each gene comparing RNC-seq and Ribo-seq. (a) HBE cell line, (b) A549, (c) MCF7. The p-value < 0.001 was obtained using the Mann-Whitney test.

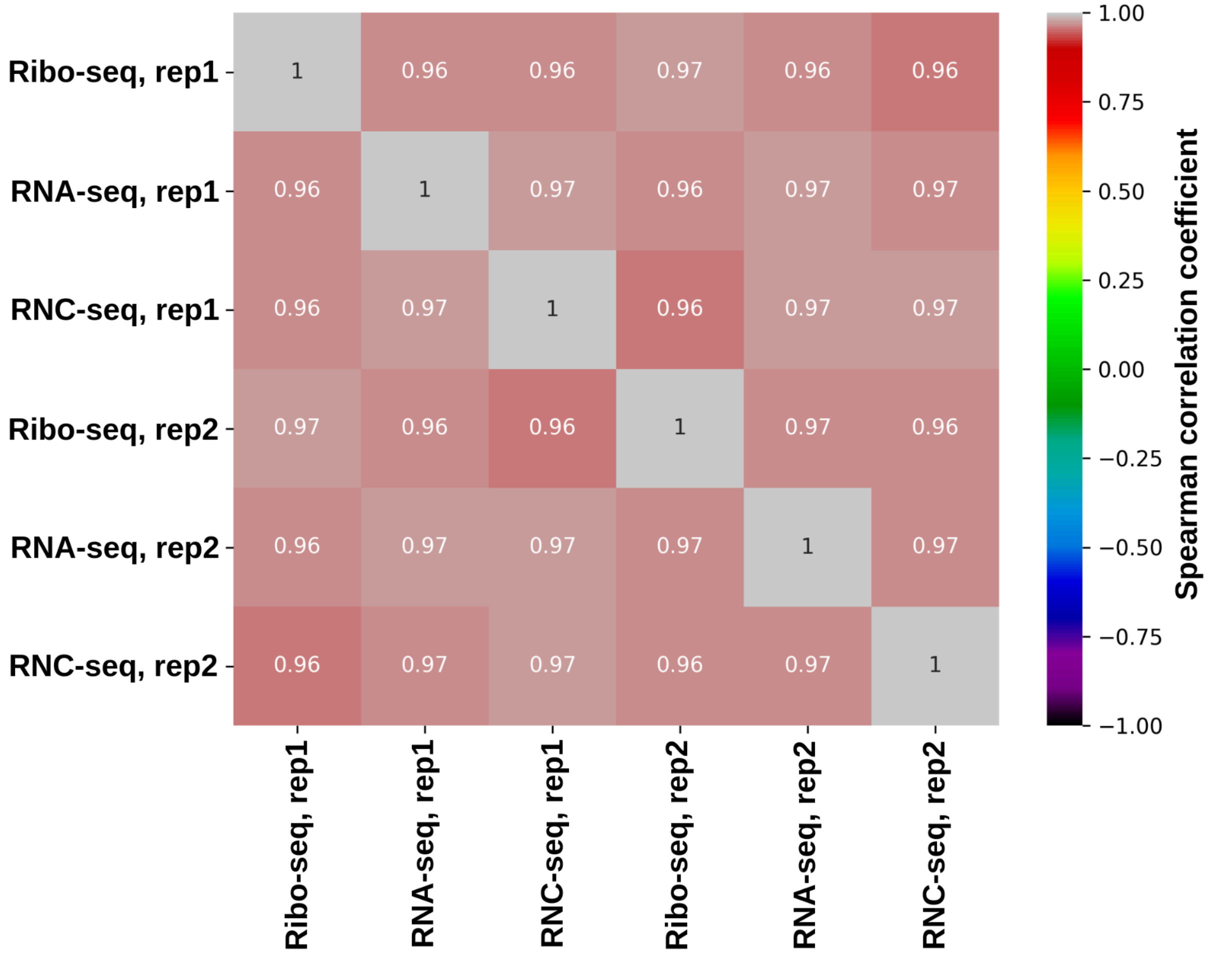

Figure S2. Spearman correlation heatmap for Ribo-seq, RNC-seq and RNA-seq. The p-value < 0.001 was estimated with 1000 permutations for each comparison.

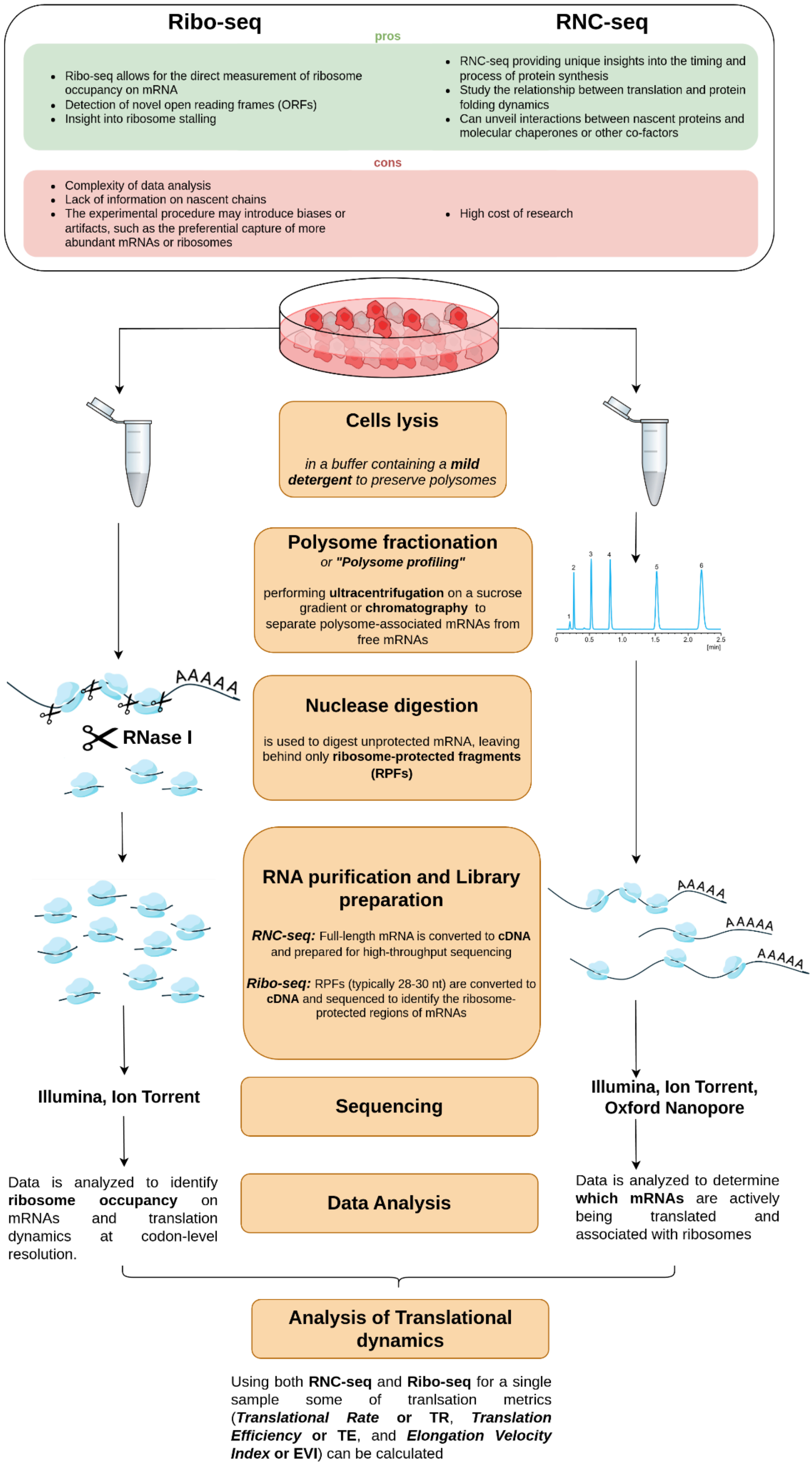

Figure S3. The comparison of Ribo-seq and RNC-seq.
